# Single‐Retainer Lithium Disilicate Resin‐Bonded Bridge for Mandibular Central Incisor Replacement Using CAD/CAM and Virtual Articulator: A 25‐Month Case Report

**DOI:** 10.1002/ccr3.72341

**Published:** 2026-03-19

**Authors:** I. Hariri, F. Dabla, A. El Yamani

**Affiliations:** ^1^ Department of Fixed Prosthodontics, Faculty of Dentistry Mohammed V University Rabat Morocco

**Keywords:** CAD/CAM, case report, lithium disilicate, mandibular central incisor, minimally invasive dentistry, resin‐bonded bridge

## Abstract

Rehabilitation of a missing mandibular central incisor with a resin‐bonded single‐retainer fixed dental prosthesis is challenging, particularly in young patients where implant placement may be contraindicated. This case describes the replacement of a mandibular left central incisor in an 18‐year‐old male following trauma, using a lithium disilicate single‐retainer resin‐bonded bridge fabricated via CAD/CAM technology and designed with a virtual articulator for occlusal optimization. A strictly enamel‐limited preparation was performed on the left lateral incisor. The prosthesis was designed and milled from lithium disilicate glass ceramic (E.max CAD) and bonded following standard adhesive protocols. Immediate integration was achieved without occlusal adjustments. At 25 months, clinical examination revealed stable periodontal tissues, absence of inflammation, and satisfactory esthetic integration. This case highlights a conservative digital workflow for anterior mandibular tooth replacement, combining lithium disilicate material with virtual articulator planning to minimize adjustments and preserve dental tissues. Long‐term studies are required to confirm durability in this indication.

## Key Clinical Message

A single‐retainer lithium disilicate resin‐bonded bridge, digitally planned and fabricated with CAD/CAM technology and verified on a virtual articulator, can offer a conservative treatment option for anterior mandibular tooth replacement in young patients. This approach may reduce intraoral adjustments and preserve tooth structure; however, long‐term follow‐up is required to confirm its durability and functional performance.

## Graphical abstract

A digital workflow for lithium disilicate resin‐bonded prosthesis enables minimally invasive rehabilitation with improved accuracy and esthetics.

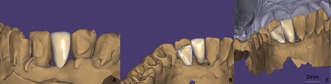

## 1 Introduction

Replacing a missing mandibular incisor is challenging due to esthetic, biomechanical, and functional demands; anterior tooth loss affects 0.2%–15.7% [1], and in young adults, resin‐bonded bridges are valuable when implant placement is contraindicated or delayed [2].

Introduced by Rochette in 1972 [3], early resin‐bonded bridges consisted of perforated metal wings bonded without tooth preparation but demonstrated high failure rates, limiting their indication for long‐term treatments [4]. In 1996, Hussey and Linden reported high survival rates for porcelain‐fused‐to‐metal single‐retainer resin‐bonded bridges [5]. One year later, Kern proposed a new design and protocol based on single‐retainer ceramic resin‐bonded bridges, improving longevity and esthetic outcomes [6]. A single‐retainer configuration is generally preferred over a two‐retainer design because differential mobility between abutment teeth may lead to debonding of the weaker abutment when two retainers are used [7]. In addition, this approach allows greater tissue preservation, reduces the risk of pulpal involvement, and facilitates oral hygiene by enabling easier passage of interdental cleaning devices under the pontic [8]. According to a systematic review conducted by Junhyun Chen et al., cantilevered all‐ceramic resin‐bonded bridges demonstrate a significantly higher survival rate (*p* < 0.01) compared with two‐retainer all‐ceramic designs [9]. Nevertheless, several complications have been reported, including debonding, minor ceramic chipping, and occasional framework fractures [9, 10].

Long reserved for specific indications, this modality has regained interest through advances in adhesive dentistry, high‐strength ceramics, and digital workflows. This case reports a mandibular lithium disilicate single‐retainer resin‐bonded bridge fabricated using CAD/CAM and a virtual articulator to replace a traumatized mandibular left central incisor in a healthy 18‐year‐old patient.

## 2 Case History/Examination

A healthy 18‐year‐old male patient with no significant medical history presented to the fixed prosthodontics department for the rehabilitation of a missing mandibular left central incisor. Both mandibular central incisors had previously undergone reimplantation following a road traffic accident. The left central incisor subsequently exfoliated, whereas the right central incisor remained in place.

The patient reported considerable psychological distress related to the anterior tooth loss. He described having temporarily interrupted his studies due to the esthetic and emotional impact of the trauma. No deleterious oral habits, parafunctional behaviors, substance use, or unfavorable dietary patterns were identified.

### 2.1 Clinical and Radiographic Findings

**FIGURE 1:**
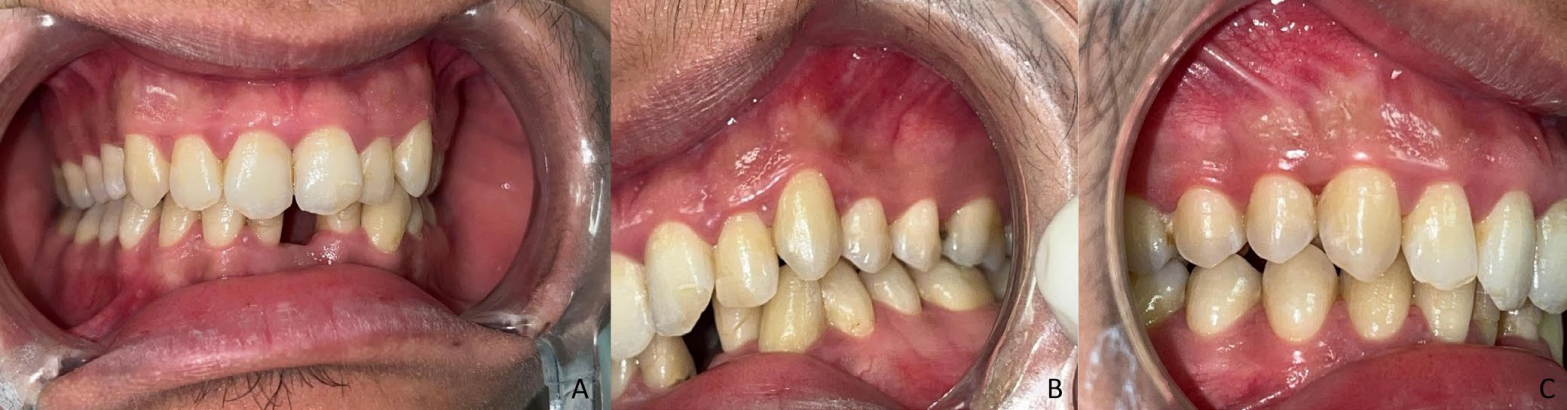
Initial clinical situation. (A) Frontal view, (B) right lateral view, (C) left lateral view. All teeth present except the mandibular left central incisor. Healthy periodontium and stable maximal intercuspation observed.

**FIGURE 2:**
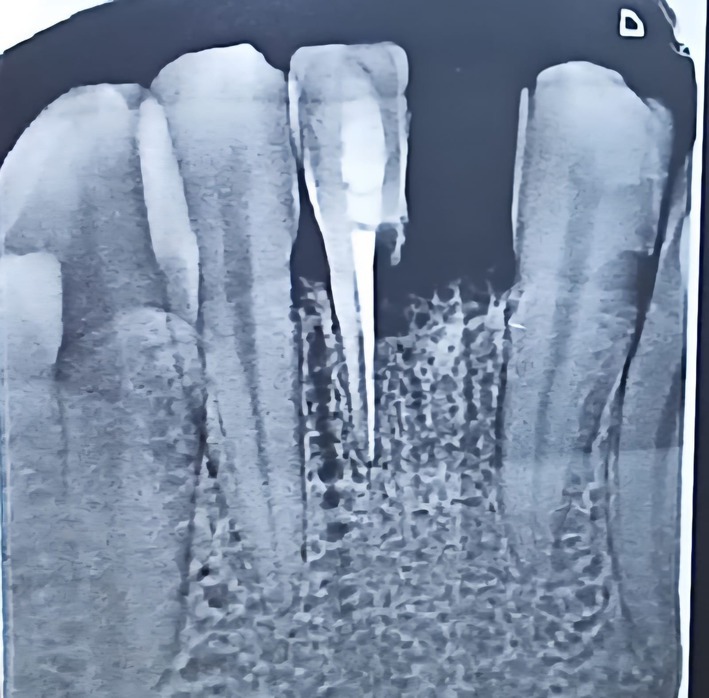
Periapical radiograph showing the edentulous mandibular left central incisor site and the reimplanted right mandibular central incisor. External root resorption consistent with no signs of periapical pathology or infection. The endodontic treatment appears satisfactory, and the alveolar ridge presents adequate bone height with no detectable abnormalities.

- Oral hygiene was satisfactory, with twice‐daily toothbrushing.
- The periodontium was healthy, with no clinical signs of gingival inflammation.
- The dentition was complete except for the missing mandibular left central incisor.
- Composite remnants were present on the labial surfaces of the left mandibular lateral incisor and the right mandibular central incisor, consistent with the previous placement of a buccal fixed retainer.
- The left mandibular lateral incisor, selected as the abutment tooth, was caries‐free, properly aligned, structurally sound, and presented sufficient enamel surface suitable for adhesive procedures.
- The edentulous ridge displayed a Cawood and Howell Class III morphology, characterized by a rounded crest with adequate height and width, covered by healthy fibromucosa.
- The patient presented a Class I Angle occlusion with preserved anterior functional guidance. Standardized intraoral photographs with lip retractors were obtained to document the baseline clinical situation (Figure 1).
- A preoperative periapical radiograph was obtained prior to treatment planning (Figure 2). The radiograph showed a healthy edentulous ridge with no signs of pathology or fracture. The right mandibular central incisor demonstrated an adequate endodontic treatment with radiographic evidence of external root resorption, consistent with the physiological resorption process observed after reimplantation following traumatic avulsion. No periapical lesions or infectious pathology were detected. The tooth was asymptomatic, non‐mobile, and is currently under periodic monitoring by an endodontic specialist for potential retreatment if required.

## 3 Differential Diagnosis, Investigations and Treatment

### 3.1 Diagnosis and Therapeutic Decision

A diagnostic wax‐up was performed following a preliminary impression, in order to preview the prosthetic outcome and confirm the spatial compatibility of the edentulous site with the dimensions of the missing tooth.

Several treatment options were discussed:

- Dental implant: Ruled out due to potential residual craniofacial growth at the patient's age and financial constraints.
- Conventional fixed dental prosthesis: Deemed excessively invasive for a case requiring a conservative approach.

After interdisciplinary consultation and informed consent, the treatment of choice was a resin‐bonded single retainer fixed dental prosthesis fabricated with lithium disilicate‐reinforced glass–ceramic (E.max CAD), in accordance with the principles of adhesive and minimally invasive dentistry. To enhance the accuracy of the prosthetic fabrication and improve treatment predictability, a digital workflow was implemented. Economically, the resin‐bonded single‐retainer fixed dental prosthesis is generally less costly than implant therapy, which requires surgery, potential grafting procedures, and multiple follow‐up visits. In fact, implant treatment is estimated to be approximately 40% more expensive than the chosen approach. Additionally, the resin–bonded single retainer fixed dental prosthesis involves fewer clinical steps, shorter treatment time, and lower overall resource use, representing a cost‐effective alternative while maintaining good esthetic and functional outcomes.

### 3.2 Preparation of the Abutment and Impression

**FIGURE 3:**
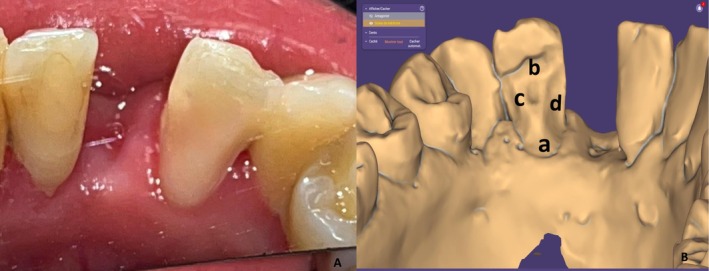
Abutment tooth preparation. (A) Clinical preparation, (B) virtual model. Labels: (a) chamfer finish line, (b) incisal limit, (c) macro‐retentive pit, (d) connector box.

A strictly enamel‐limited preparation of the abutment tooth (the left lateral mandibular incisor) was performed. A 1 mm thickness chamfer margin (a) in the lingual surface was placed in a supragingival position using a chamfer bur. The occlusal limit of the preparation (b) was placed 2 mm from the incisal edge to preserve the translucency of the incisal edge. This limit was created using an olive‐shaped bur held perpendicular to the incisal edge, with a depth of 1 mm. A lingual reduction of 1 mm was carried out with the same bur parallel to the lingual surface. A 1 mm deep macro‐retentive pit (c) was created with a round bur to aid in the accurate repositioning of the prosthetic component (Figure 3).

A connector box measuring 12 mm^2^ (4 mm height × 3 mm width) (d) was prepared using a round bur to meet the mechanical requirements of the ceramic material and prevent future connector fractures (Figure 3). After finishing the preparation, the following criteria should be present in the preparation of our abutment:

- Chamfer as a finish line with 1 mm thickness in supragingival position in the lingual surface
- A connector box of 12mm2 (4 mm width × 3 mm height) (millimeters)
- 1 mm Reduction of the lingual surface
- 1 mm deep macro retentive pit

**FIGURE 4:**
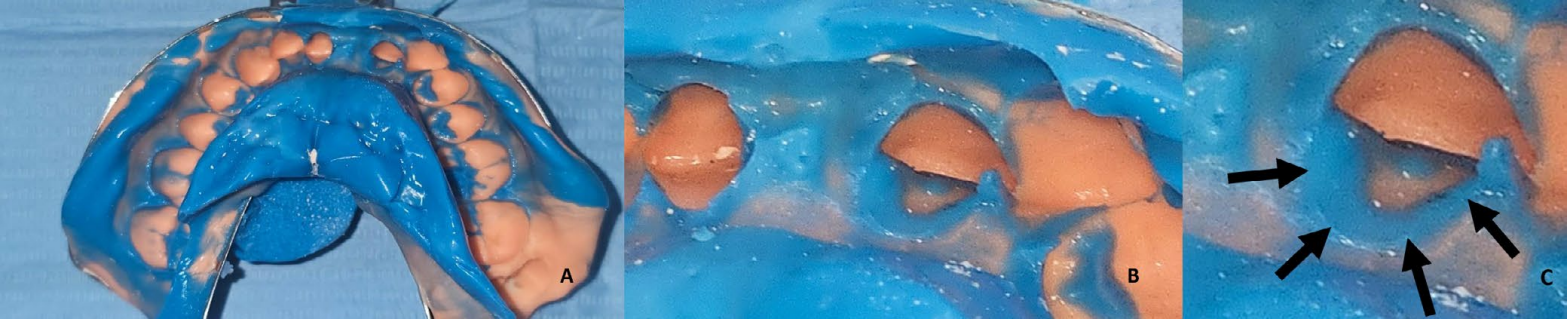
Final impression with polyvinyl siloxane. (A) Full impression, (B) detailed visualization of preparation margins within the impression material, (C) A macro‐image highlighting the finish line details, with black arrows indicating the area of interest.

Following validation of the preparation, a single‐step impression was taken using polyvinyl siloxane (Figure 4).

### 3.3 Computer Aided Design/Manufacturing Steps

**FIGURE 5:**
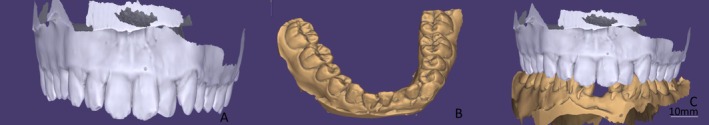
Digital scanning of models. (A) Maxillary arch, (B) Mandibular arch, (C) occlusion.

The CAD/CAM (Computer‐Aided Design and Computer‐Aided Manufacturing) process was initiated once the working and the antagonist casts were obtained. Prior to data acquisition, the scanner was calibrated according to the manufacturer's instructions using the supplied calibration tool to ensure optimal measurement accuracy. Scanner accuracy was verified by scanning a standardized calibration object provided by the manufacturer and comparing the obtained data with the reference file to confirm dimensional consistency within the tolerance range (< 20 μm). After calibration, the scanning of the working model and the antagonist, as well as the occlusion, was performed using a laboratory scanner (Figure 5).

**FIGURE 6:**
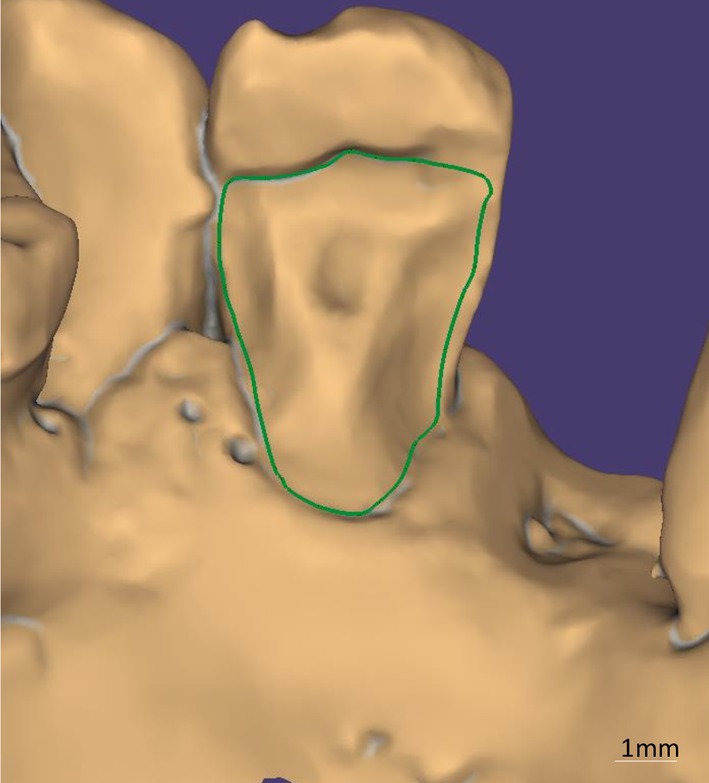
Delineation of the finish line (green). Manual adjustment used instead of automatic software detection to improve accuracy.

**FIGURE 7:**
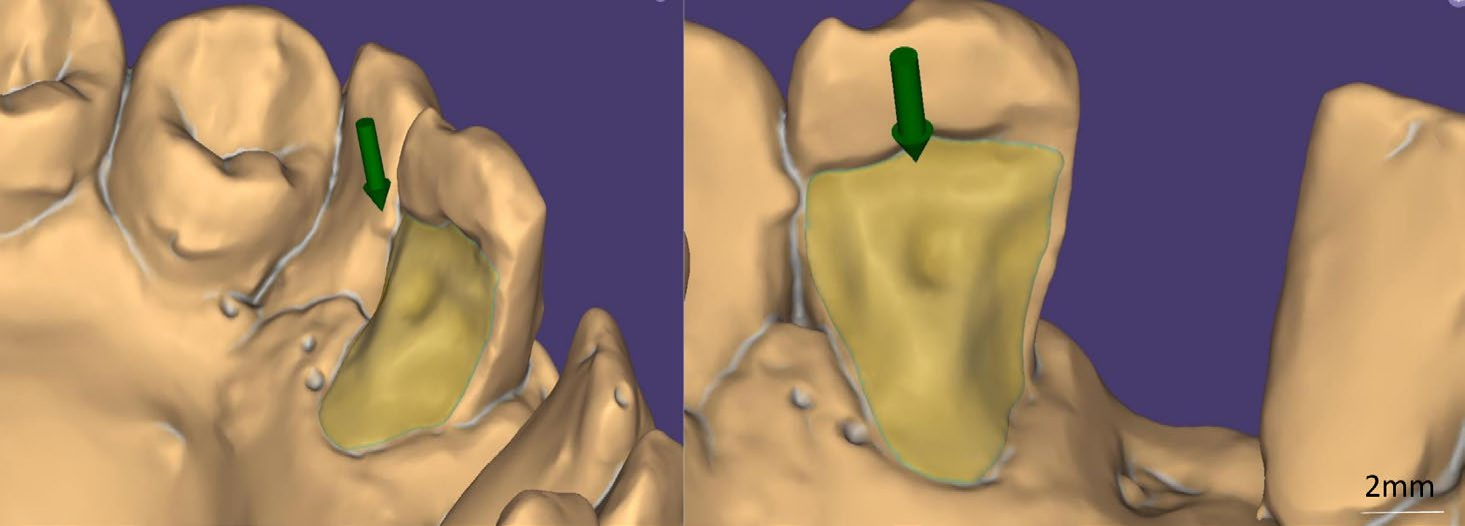
Determination of insertion axis (green arrow) and design of intaglio surface (yellow).

**FIGURE 8:**
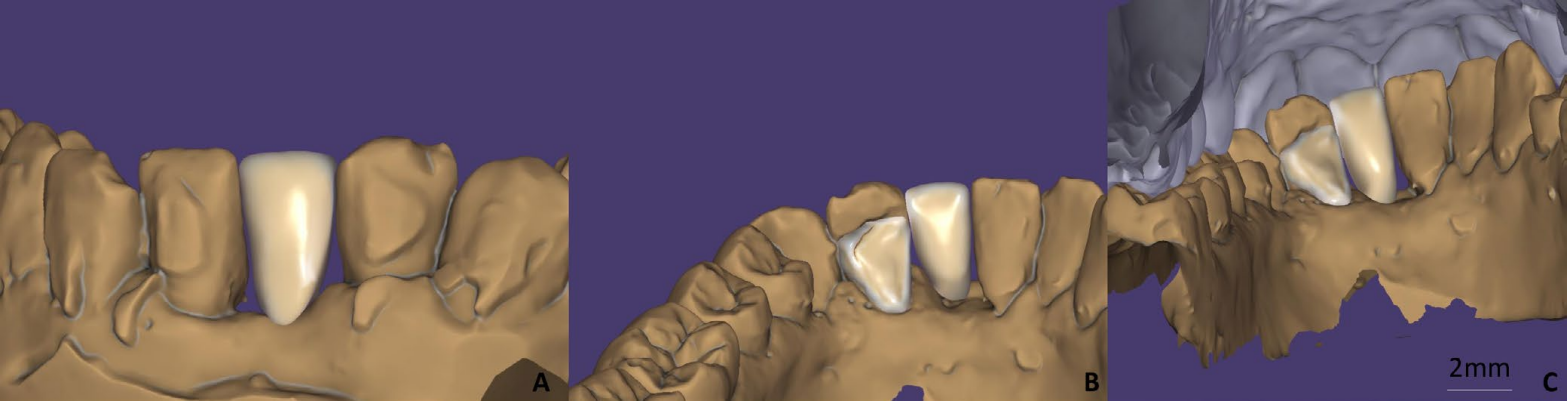
Pontic design in multiple planes. (A) Buccal view, (B) lingual view, (C) relation to opposing dentition.

The Computer‐Aided Design (CAD) phase comprised several sequential steps:

Manual delineation of the finish line (marked in green) was chosen over the software's automated suggestion to ensure precise position (Figure 6). Following the definition of the insertion axis, the design of the intaglio surface of the retainer and its external contour was conducted (Figure 7). Subsequently, the design of the prosthetic intermediate was carried out, with a three‐dimensional analysis to assess its adaptation to the edentulous ridge, adjacent teeth, and the opposing dentition (Figure 8).

**FIGURE 9:**
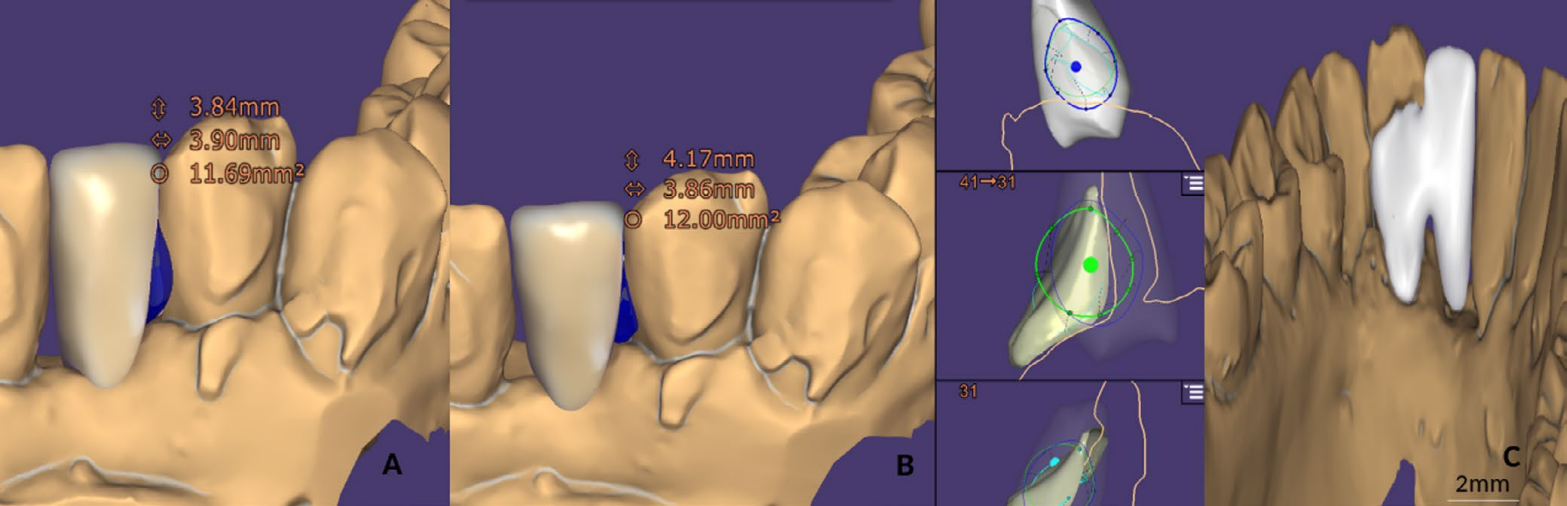
Connector design in Exocad. (A) Inadequate surface area of 11.60 mm^2^, (B) corrected surface area of 12 mm^2^, (C) lingual morphology.

**FIGURE 10:**
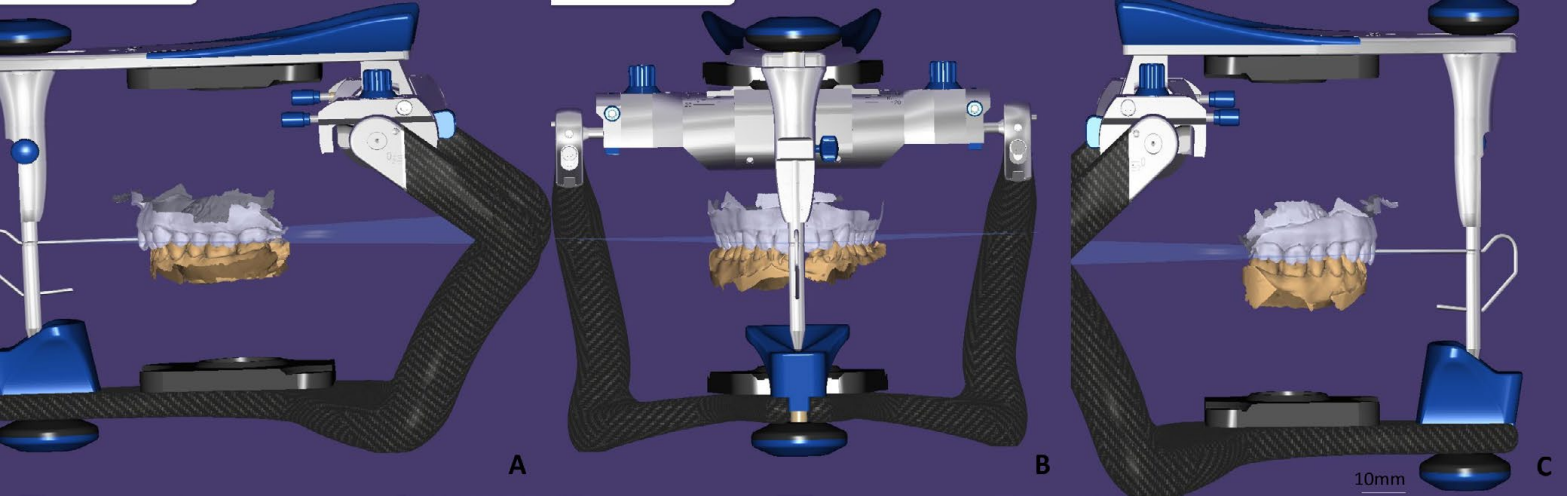
Virtual articulator mounting using average condylar values. (A) Right view, (B) frontal view, (C) left view.

**FIGURE 11:**
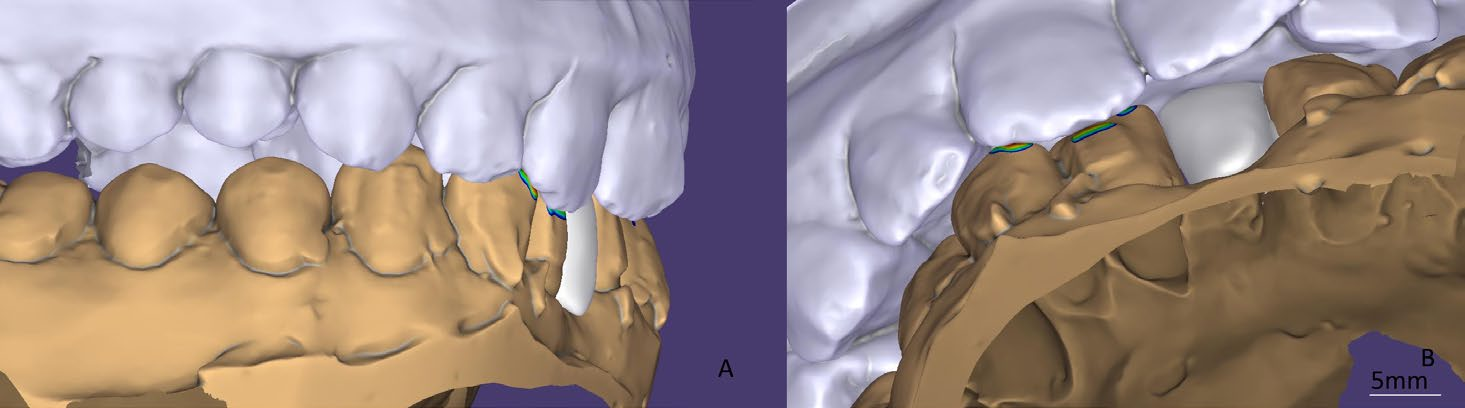
Dynamic occlusion analysis in virtual articulator. (A) Protrusion, (B) lateral excursion. Pontic confirmed free of contact in all functional movements.

The design of the connector, a key element for the mechanical durability of the structure, was carried out in compliance with the minimum cross‐sectional area of 12 mm^2^, as recommended for glass–ceramics. The use of Exocad software and its “multi‐view” function allowed for a detailed multidimensional analysis of the connector thickness, thereby minimizing the risk of unexpected material fracture (Figure 9). The models were virtually mounted on an articulator using average condylar values, facilitating the optimization of static and dynamic occlusal contacts (Figure 10). As recommended by Mathias Kern, the pontic was excluded from all dynamic occlusal contacts, a finding confirmed through virtual articulator analysis (Figure 11).

Following the completion of the digital design, the milling procedure was executed using a 5‐axis milling machine, controlled by CAM (computer‐aided manufacturing) software, and employing lithium disilicate glass–ceramic blocks (E.max CAD type) as the restorative material. Concerning the milling parameters, two burs were used during the procedure. The details regarding bur diameter, material, spindle speed, feed rate, and milling mode are presented as follows:

Coarse bur:

- Bur diameter: 1.6 mm (millimeter)
- Material: Diamond‐coated
- Spindle speed: 50,000 rpm (Revolutions Per Minute)
- Feed rate: 600 mm/min (millimeter/min)
- Milling mode: Wet

Fine bur:

- Bur diameter: 1.0 mm
- Material: Diamond‐coated
- Spindle speed: 60,000 rpm
- Feed rate: 400 mm/min
- Milling mode: Wet

These parameters were selected in accordance with the manufacturer's recommendations to ensure optimal surface integrity, marginal accuracy, and to minimize the risk of microcracks during lithium disilicate milling.

### 3.4 Bonding Protocol

**FIGURE 12:**
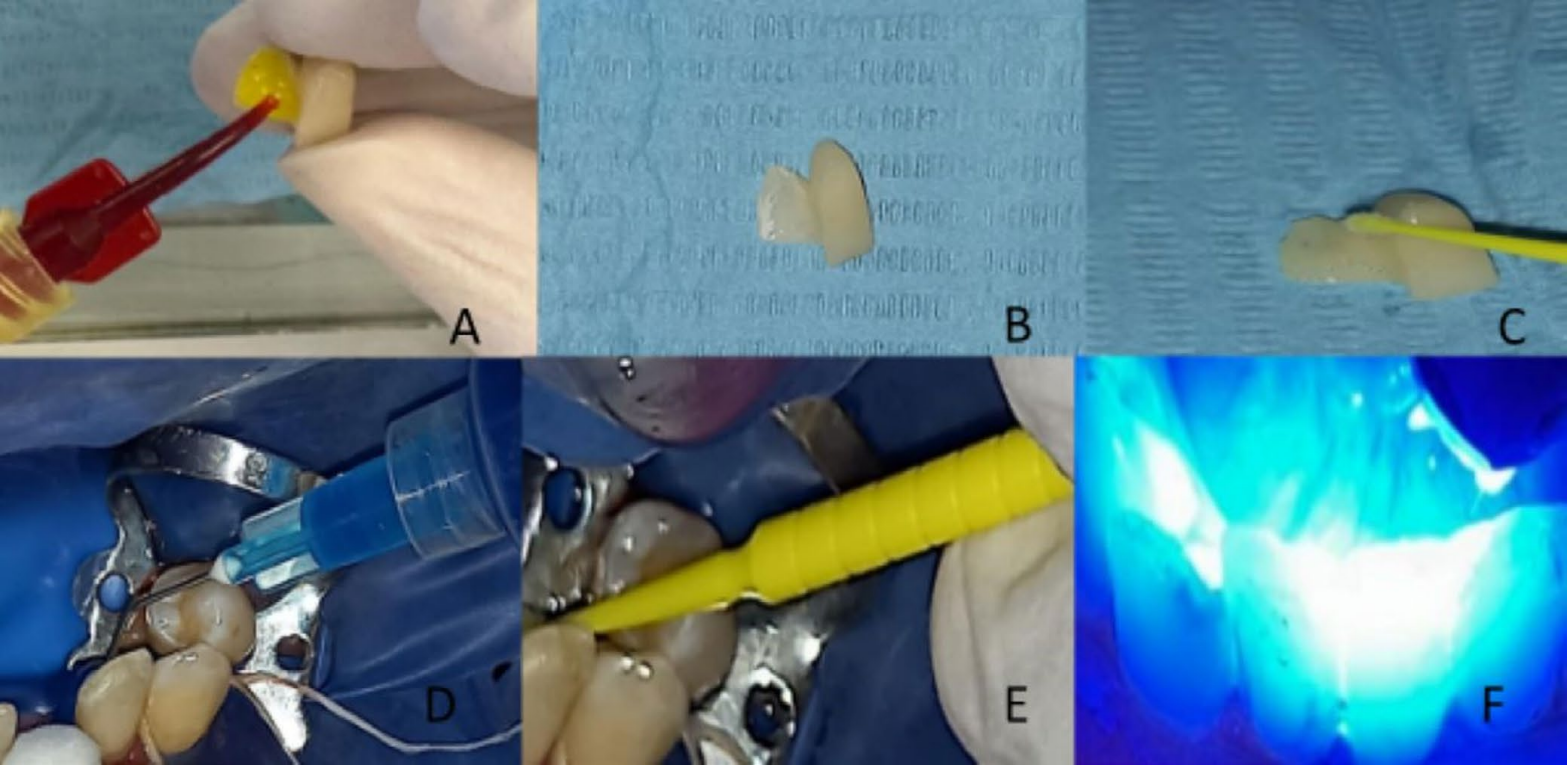
Bonding protocol. Prosthesis: (A) hydrofluoric acid 5% etching, 30 s, (B) rinsing and drying, (C) silane application, 2 min. Tooth: (D) 37% phosphoric acid etching, 30 s, (E) adhesive application and light curing, 20 s, (F) resin cement application and light curing, 20 s per surface, repeated three times.

After obtaining the prosthesis, a try‐in appointment allowed verification of esthetics and integration of the prosthesis. Once the approbation of the patient was gained, the bonding protocol was carried out under rubber dam isolation; the intaglio surface of the prosthesis was etched with 5% hydrofluoric acid for 30 s, rinsed, air‐dried, and silanized for 2 min (Figure 12).

The tooth surface was treated by etching with 37% phosphoric acid for 30 s, followed by thorough rinsing and drying. A universal adhesive was then applied and light‐cured for 20 s. The restoration was cemented using a dual cure adhesive resin cement. Excess cement was removed, and light curing was performed for 20 s per surface, repeated three times to ensure optimal polymerization (Figure 12). All procedures were conducted in a controlled clinical environment with an ambient temperature of 22°C–24°C (Celsius) and relative humidity of 45%–55%. These parameters were selected based on the manufacturer's recommendations and evidence that resin cement polymerization and bond strength are sensitive to environmental conditions. Maintaining this temperature ensures predictable adhesive handling and optimal polymerization, while controlling humidity minimizes moisture contamination and prevents porosity or compromised adhesion. By standardizing these environmental conditions, bonding reliability and clinical reproducibility are maximized.

## 4 Outcome and Follow Up

**FIGURE 13:**
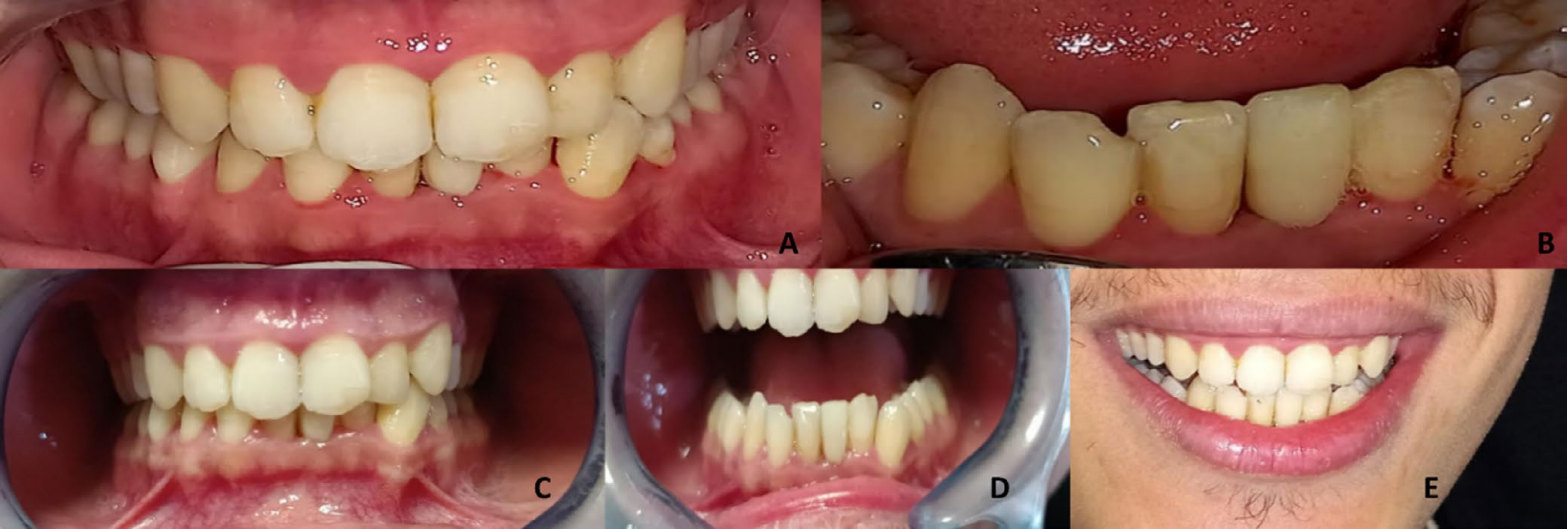
Clinical outcome. (A, B) Immediate integration after bonding. (C, D) 25 month follow‐up showing stable periodontal tissues and satisfactory esthetic integration. (E) Patient's smile demonstrating restored dental harmony.

The immediate integration was evaluated after rubber dam removal, and no occlusal adjustments were necessary, thanks to the virtual articulator planning (Figure 13).

A 25 months follow‐up was conducted, showing an optimal biological integration with stable marginal tissues with no inflammation or infection; the following periodontal parameters were evaluated to confirm this periodontal tissue stability:

- Probing depth: All sites around the abutment tooth measured ≤ 3 mm.
- Gingival index: Loe and Silness Gingival Index was used; it was quantified 0 indicating normal gingiva, no inflammation and no bleeding.
- Width of keratinized gingiva: Maintained at 5 mm around the restoration margins.
- Mobility of the abutment tooth: Assessed as Miller Class 0, indicating no detectable mobility.

The esthetic integration of the prosthetic reconstruction was also observed. The patient did not express any discomfort and regained a harmonious confident smile (Figure 13).

## 5 Discussion

Single‐unit anterior tooth loss, particularly in young adults, presents a complex challenge that is simultaneously functional, biological, and esthetic. This complexity arises from the critical role of the anterior region in smile esthetics, phonetics, and occlusal guidance, as well as the significant psychological and social impacts associated with tooth loss in this area [11]. Therapeutic options for anterior edentulism include dental implants, conventional fixed partial dentures, removable prostheses, and resin‐bonded bridges.

Among these, the resin–bonded single retainer fixed dental prosthesis fabricated from glass–ceramic is a minimally invasive and conservative treatment option, particularly indicated in young patients where residual craniofacial growth often contraindicates implant placement. Residual growth is well documented in the literature and poses specific risks: infraocclusion of the implant restoration in the maxilla and rotational displacement of the implant in the mandible. Two systematic reviews [12, 13] have highlighted these complications. The resin‐bonded single retainer bridge offers the advantage of maximal tissue preservation, avoiding extensive preparation and preserving pulp vitality [14]. The choice of lithium disilicate as the restorative material is supported by its excellent translucency, esthetic integration, biocompatibility, and adhesive properties. Although its flexural strength (360–400 MPa) is lower than that of zirconia, it remains sufficient for anterior single retainer–bonded bridge indications, especially when combined with an optimal bonding protocol [15, 16, 17]. In contemporary restorative dentistry, lithium disilicate and zirconia are among the most widely used ceramic materials, each presenting specific advantages and limitations. Lithium disilicate offers excellent optical properties and adequate mechanical strength, providing predictable esthetic integration and functional performance. It demonstrates high biocompatibility, favorable abrasiveness against opposing dentition, and reliable marginal accuracy. Its ability to be etched enables strong adhesive bonding, supporting both monolithic and layered restorations. However, intraoral adjustments are technique sensitive, veneering ceramic chipping may occur, and glazing or fluorapatite layering can increase antagonist wear.

Zirconia exhibits superior mechanical strength and outstanding fracture resistance, combined with acceptable optical properties. It is highly biocompatible, shows low plaque accumulation, and offers stable marginal and internal fit. Nevertheless, zirconia is more opaque than glass ceramics, cannot be etched using conventional protocols, limiting adhesive bonding, and may undergo low temperature degradation. Careful finishing and glazing are required to minimize wear clinically effectively [18].

Concerning the complications associated with single–retainer fixed resin‐bonded bridges, Miettinen and Millar reviewed several clinical follow‐up studies evaluating the performance of all‐ceramic resin‐bonded bridges, including single‐retainer designs. Their findings indicated that bridge fractures and debonding were the primary causes of failure [19]. Moreover, cantilever resin‐bonded bridges present several inherent limitations. Successful outcomes depend strongly on appropriate case selection, particularly the absence of parafunctional habits. A meticulous bonding protocol and excellent patient oral hygiene are also critical to ensure long‐term success. In the event of debonding, clinical management can be challenging and may necessitate complex repair procedures as well as close postoperative monitoring. Furthermore, limited access to advanced CAD/CAM technology in certain clinical environments may restrict the widespread adoption of this restorative approach in routine dental practice [20]. The preparation design followed the guidelines denounced by Mathias Kern: a chamfer finish line with 1 mm supragingival depth for glass‐ceramics and 0.6 mm for zirconia, with a 2 mm incisal clearance to preserve incisal translucency. Additionally, a macro‐retention groove was included to facilitate accurate repositioning and improve mechanical retention. The connector dimensions were adapted to material type: 12–16 mm^2^ for lithium disilicate and 6 mm^2^ for zirconia [21, 22]. Numerous studies have confirmed the long‐term reliability of this prosthetic solution. In a longitudinal study, Kern [23] reported a 10‐year survival rate exceeding 95.4% and 81.8% at 18 years. Similarly, Saker and al. reported survival rates ranging from 90% to 100% over 60 months [24]. The present case is notable for its use of a lithium disilicate single retainer resin‐bonded bridge to replace a mandibular central incisor, in conjunction with a digital workflow, including computer‐aided design and manufacturing (CAD/CAM) and virtual articulator integration for enhanced occlusal control. Several biomechanical considerations must be carefully evaluated before initiating a single‐retainer restoration; one key factor is the reduced enamel bonding surface, particularly in small mandibular anterior teeth, which may predispose the adhesive interface to higher stress concentrations. A finite element study in similar configurations has consistently demonstrated increased stress accumulation at the retainer–cement interface under horizontal loading, especially in cantilevered designs [25].

Another critical mechanical factor is the dimension of the future connector. Another finite element study has highlighted that the base of the connector significantly influences the fracture strength of the prosthesis. Regardless of connector shape, a sufficiently sized base is essential to resist crack propagation and prevent material failure. Conversely, a small connector base can induce greater tensile stress on the lingual face of the prosthesis. Furthermore, when connector height is altered while maintaining a fixed width, increased stress is imposed on the prosthesis, emphasizing the importance of both height and width in load distribution [26].

The integration of digital technologies significantly improved the design phase, particularly through the use of the virtual articulator, which enabled accurate simulation of mandibular movements [27, 28]. This allowed for the identification and elimination of occlusal interferences, validating the pontic's occlusion prior to clinical placement and reducing intraoral adjustments. Moreover, precise three‐dimensional management of the connector design plays a critical role in preventing premature fracture.

It is also important to emphasize that digitally fabricated restorations demonstrate improved marginal adaptation and fit compared to conventional techniques [29].

The bonding protocol, as with all indirect restorations, was adapted to the restorative material. In this case, the lithium disilicate framework was etched with hydrofluoric acid for 30 s, rinsed and dried, followed by application of a silane coupling agent, and bonded using a dual‐cure adhesive resin cement, ensuring strong micromechanical and chemical bonding between the ceramic and tooth structure [30, 31]. This protocol is essential for restoration longevity. Limitations of this case include the relatively short follow‐up period of 25 months, which does not yet allow for evaluation of long‐term biomechanical and periodontal stability. Further prospective studies with larger cohorts are required to validate the promising outcomes of this conservative approach in challenging mandibular anterior indications.

## 6 Conclusion

A single‐retainer lithium disilicate resin‐bonded bridge designed using CAD/CAM and an articulator enabled conservative mandibular incisor replacement, minimized adjustments; however, 25‐month outcomes require longer term evaluation.

## Author Contributions

**I. Hariri:** conceptualization, resources, validation, writing – original draft, writing – review and editing. **F. Dabla:** writing – review and editing. **A. El Yamani:** writing – review and editing.

## Funding

This research received no external funding.

## Consent

Written informed consent was obtained from the patient to publish this report in accordance with the journal's patient consent policy.

## Conflicts of Interest

The authors declare no conflicts of interest.

## Data Availability

Data related to the study can be provided on reasonable request.
